# Exploring teacher design team endeavors while creating an elementary-focused STEM-integrated curriculum

**DOI:** 10.1186/s40594-017-0084-1

**Published:** 2017-10-06

**Authors:** Justin R. McFadden, Gillian H. Roehrig

**Affiliations:** 1 University of Louisville, Louisville, KY 40292 USA; 2 University of Minnesota, St. Paul, MN 55108 USA

**Keywords:** Teacher design teams, Curriculum design, STEM education, Elementary education

## Abstract

**Background:**

This study presents two teacher design teams (TDTs) during a professional development experience centered on science, technology, engineering, and mathematics (STEM)-integrated curriculum development. The main activity of the study, curriculum design, was framed as a design problem in order to better understand how teachers engaged with the complexities of integrated curriculum development. Additionally, Remillard’s, (Review of Educational Research 75:211–246, 2005) teacher-curriculum “participatory relationship” provided a framework for further exploring teacher actions during the design process. Utilizing a case study research design, participant curriculum design conversations were audio-recorded for 12 days during a summer professional development experience. Constructed grounded theory and a method of selective coding revealed insights about the processes and supports that enable collaborative curriculum design.

**Results:**

Results showed that when a TDT is not prompted and/or enabled to first lay out and articulate the overall value of a STEM-integrated curriculum, they will rightly follow their intuitions as classroom teachers and engage in the process accordingly. Second, involving practicing teachers in the curriculum design process requires complete “participation” with the curriculum ideas they are contemplating because in the end, the curriculum’s resultant lessons will be taught in their own and other teacher’s classrooms.

**Conclusions:**

The findings from this study indicate the importance of “pushing” active classroom teachers from the design to the mapping arena by instituting curriculum development activities and/or strategies (i.e., processes) that might help a TDT develop a “voice” (Remillard, From Text to 'Lived' Resources:105-122, 2011) or “value” (Dorst, Design Studies 22:4–17, 2006) for the curriculum under development. If members of a TDT are willing to reveal their interpretations, perceptions, and beliefs about the conceptual ideas embedded within the curriculum being developed, both the teachers and curriculum being developed will benefit. Finally, teachers should be made aware of their roles and responsibilities, beyond superficial descriptions; and understand participation in STEM-integrated curriculum design brings with it the likelihood their individual ideas, perceptions, and beliefs will be integrated within the curriculum being developed.

**Electronic supplementary material:**

The online version of this article (10.1186/s40594-017-0084-1) contains supplementary material, which is available to authorized users.

## Background

Curriculum is a central tenet of any national call for educational reform in the USA (Powell and Anderson, 2002). Science curriculum developed in the USA in the 1960s was primarily lead by university-based scientists who instilled a method of teaching science aligned with the “logical structure of the disciplines” (Deboer, 2014; p. 564) with science being portrayed as experimental in nature (Atkin and Black, 2007). Curriculum was primarily written to limit a classroom teacher’s ability to make changes to it (Atkin and Black, 2007), which eventually resulted in teacher resistance (Fullan and Hargreaves, 1992) and instruction that matched existing, traditional practice (Apple, 1990; Arias, Bismack, Davis, and Palincsar, 2016; Ball and Cohen, 1996; Olson, 1981). As a result, researchers and curriculum developers made changes accordingly once they understood the important role teachers play when ushering in curriculum change (Brown and McIntyre, 1978; Kelly and Staver, 2005; McIntyre and Brown, 1979; Parke and Coble, 1997; Penuel and Gallagher, 2009; Remillard, 2005; Shawer, 2010; Voogt et al. 2015). As Ben-Chaim, Joffe, and Zoller (1994) state we now know, “the successful implementation of an innovative curricular program is dependent on the full active participation of the teachers involved in the decision-making process associated with the curriculum reform” (p. 365). Following this period of “teacher proof” curriculum and calls for fidelity of curricular implementation (O’Donnell, 2008; Penuel and Means, 2004), teachers now take on the dual roles of curriculum designer and curriculum implementer (Connelley and Clandinin, 1988; Eggleston, 1980; Karplus, 1971; Kelly, 1999; Penuel and Gallagher, 2009; Prawat, 1993; Stenhouse, 1975). Including teachers in curriculum development gives them a sense of ownership (Bakah, Voogt, and Pieters, 2012; Carlgren, 1999; Voogt et al. 2015) that can counteract “top-down” aversion (Fullan and Hargreaves, 1992).

And while ownership and knowledge of reform efforts aids teachers’ transition of broad-scale reform into classroom practice, their involvement can also be problematic. As Pintó (2005) noted, “Give(n) too much direction, teachers lose any sense of ownership. Give(n) too little, and they feel that they do not know what to do” (p. 2). Thus, there needs to be a balanced approach when involving and supporting teachers in the curriculum development process. Teacher involvement can be stymied by countless issues; most notably that localized contexts (e.g., material availability) overshadow teacher priorities during the curriculum design process (Boschman, McKenney, and Voogt, 2014; Davis, Beyer, Forbes, and Stevens, 2011; Joyce, 1978; Kerr, 1981; Taylor, 1980). Put another way, it is difficult to harness localized knowledge for more generalizable use (Boschman, McKenney, and Voogt, 2014) when creating “teacher design teams” [TDTs] (Huizinga, Handelzalts, Nieveen and Voogt, 2014). Curriculum design requires a certain expertise (Brown, 2009; Forbes 2009, Huizinga 2009, Nieveen and Van der Hoeven, 2011) one cannot simply assume teachers possess (Kerr, 1981).

There is a need to better understand how classroom teachers’ intricate and detailed knowledge of classrooms can be utilized as they design curricular units for more generalizable classroom use. Whether they are asked to develop curriculum in large-scale, nationally funded or district initiated, curriculum-writing projects; or creating and sharing curricular resources on commercially backed websites (e.g., https://www.teacherspayteachers.com/), it is due time to acknowledge and investigate this emerging, teacher-centric phenomenon.

### Literature review

#### National reforms for localized use

Initially spurred by the National Defense Education Act (in 1958), and later by a report from the National Commission on Excellence in Education (1983), new science and mathematics curricula in the USA has always been considered a way to ensure economic success and increase national security (NRC, 2006). Educational reform efforts posited that “national goals” and “curriculum frameworks” could be used as “guides that state and local officials might use in developing curricula for local use” (National Science Board, 1983; p. 41). The original intent was not to create a national curriculum, but rather to offer guiding frames that curriculum could be designed around.

In the following decades, the national science education standards (NRC, 1996; 2000) were created and followed with the creation of standards-aligned curriculum from national publishers and projects funded by the *National Science Foundation*. Two problematic issues were eventually identified. First, being “aligned” was interpreted very broadly, and second, no distinguishable approach to curriculum writing was identified or widely used (DeBoer, 2014). Science academic standards soon became performance-based (Krajcik, McNeill, Reiser, 2008), which further confounded the problem. This issue has come full circle with the *Next Generation Science Standards* [NGSS], which contain “performance expectations” that posit students understand and apply a particular practice within content driven contexts (NGSS Lead States, 2013). Calls for the creation of “model science, technology, engineering, and mathematics [STEM] units” (Bybee, 2010) aligned to the NGSS are intended for multiple audiences, including teachers in the field.

#### Professional development via curriculum development

Arguments that local communities and therefore teachers should determine how and what their students learn have led to integrated professional development [PD] models wherein teachers learn while developing curricular units. Combining teacher PD and curriculum development creates an interdependent activity (Shawer et al. 2008) centered on teacher practice (Clarke and Hollingsworth, 2002) where curriculum is created and teacher learning is promoted (Ball and Cohen, 1996; Elmore and Burney, 1999; Garet et al. 2001; Putnam and Borko, 2000). And while some have advocated for an *adaption* model regarding curriculum (Debarger, et al. 2017; Penuel and Gallagher, 2009), Davis et al. (2011) notes the combination of PD and curriculum development is mutually beneficial because, “teachers have localized knowledge of their students, curricular goals, and affordances and constraints of their particular professional contexts…(and)…can provide unique, on-the-ground insight into the enactment of specific curriculum materials” (p. 798). Bringing teachers together to design curriculum is commonplace, but still under researched (Voogt, Pieters, and Handelzalts, 2016).

#### Teacher design teams

Teacher design teams began in the Netherlands via two foundational dissertations (Huizinga, 2009; Handelzalts, 2009) and a plethora of interrelated studies (Boschman et al. 2014; Huizinga, 2009; Huizinga et al. 2014; Kelly and Staver, 2005), which identify professional learning as a common benefit of TDTs (Voogt et al. 2011). Because curriculum design engages TDTs in practice-orientated conversations, research has begun examining the nature and process of “expertise” (Peercy, Martin-Beltrán, Silverman, and Daniel, 2015) exhibited while engaged in the design process. *Design expertise* (Huizinga, 2009, Huizinga et al. 2014) has been used as an all-encompassing phrase that is used to describe a curriculum designer’s ability to enact the skills of analysis, design, development, implementation, and evaluation (Molenda, 2003). A knowledgeable facilitator (Becuwe et al. 2015; Nieveen and Van der Hoeven, 2011) or coach (McFadden, 2015; Binkhorst et al. 2015) typically supports novice designers to counteract the potential pitfalls many beginning designers experience by providing guidance in the form of purposefully posed questions (Binkhorst et al. 2015). As Voogt et al. (2015) note, when teachers collaborate with one another and a facilitator during the curriculum design process they, “share knowledge, exchange perspectives and tap into each other’s expertise” (p. 262). Unfortunately, as Remillard (2011) notes, “we understand little about the processes through which teachers might learn to engage with curriculum resources in substantially new ways and position themselves as partners” (p. 121) rather than just consumer.

Therefore, while we recognize the benefits of involving teachers in the development of curriculum (Voogt et al. 2011) there is still a need to examine the process itself as opposed to the outcomes alone (e.g., teacher learning). Closely examining the discussions and curriculum design decisions of two TDTs during an extended PD experience while they create a STEM-integrated curriculum is therefore a logical and appropriate method to learn more about what transpires during this process.

#### Research questions

The current study investigated how and in what manner (i.e., the *process*) two in-service, elementary TDTs created a STEM-integrated curriculum. The aim was to investigate the effects of teachers’ involvement in the development of an elementary-based, STEM-integrated curriculum. In addition to exploring the challenges a pair of TDTs encountered during the curriculum design process, the study also sought to uncover the *supports* that further enabled successful collaborative design. The following research questions guided this study:

1. What processes of collaborative curriculum design enable teacher design teams to develop a STEM-integrated curriculum?

## How do curriculum design supports help teacher design teams develop a STEM-integrated curriculum?

### Theoretical framework

#### Arenas of curriculum development

Members of a TDT are first and foremost engaged in STEM-integrated curriculum development as teachers. Remillard’s (1999) conceptualization of a “three arena model” can therefore be used to explore teacher actions and behaviors during the curriculum development process. The focus of this study only includes the *design* and *mapping* arena wherein the “design arena” involves the selection and/or creation of activities for immediate classroom use while the “mapping arena” involves more large-scale unit planning. In addition to selecting and creating resources for classroom use, large-scale unit planning also requires one to make decisions about content scope and sequence, something teachers do less often (Pintó, 2005).

On a day-to-day basis, teachers normally operate within the design arena (Remillard, 1999). Teachers typically spend a majority of their time working out the details of a curriculum idea quickly after inception (Koh, Chai, Wong, and Hong, 2015), which, given the affordances and constraints of each arena, can potentially be disadvantageous. In the *design arena*, teachers modify existing lessons for upcoming classroom use (i.e., “lesson planning”). The *mapping arena* is categorically different from the design arena, mainly because it “is not directly related to daily, classroom events; rather, it impacts and is impacted by them” (Remillard, 1999; p. 322). Within the mapping arena, teacher’s actions shift and they begin to view the curriculum holistically as a “system” of individual lessons (Reigeluth and Avers, 1997) that align to a previously determined and desired goal as a curriculum developer would.

Within the mapping arena, teachers must balance tensions between goals and constraints because developing STEM-integrated curriculum requires them to embark on “a sequence of decisions…to balance goals and constraints” (Edelson, 2001, p. 108). Tensions arise within the mapping arena, or design problem space (Goel and Pirolli, 1992), because one must remain committed (Jonassen, 2000) and exhibit a “tolerance for discomfort” (Remillard, 2005; p. 229) as decisions get made and new ideas get refined. Ultimately, teachers face a “design problem” (Jonassen, 2000, 2011) when emerged in the mapping arena because the task is ambiguous and ill structured. Within the mapping arena, there is no predetermined solution path to follow. Additionally, it necessitates the integration of multiple knowledge domains (Jonassen, 2000). Given the nature of the task and the past experiences of the participants, both the design and mapping arenas are useful concepts for examining the conversations that emerge when a TDT develops a STEM-integrated curriculum.

#### Participating with the curriculum being designed

A “participatory relationship” emerges when teachers are involved with the selection, interpretation, creation, reconciliation, accommodation, and modification of curricular resources (Remillard, 2005). Remillard’s (2005) teacher-curriculum framework “reflects a comprehensive view” (Davis, Jansen, and Van Driel, 2016; p. 2) of this relationship that accounts for the ensuing interactions that emerge when teachers and curriculum come together. The interactions that emerge during the aforementioned activities (e.g., curriculum accommodation) are encompassed within either the design or mapping arena (Remillard, 1999). As teachers engage with the ideas and suggestions of a given curriculum, they “draw on their own resources and capacities to read, make meaning of, evaluate, adopt, adapt, and replace the offerings of the curriculum” (Remillard, 2005; p. 234) in a participatory manner.

The foundational ideas and assumptions of a curriculum become actualized within this give-and-take relationship because both components of the framework (i.e., teacher and curriculum) will eventually come together at a given point within the classroom. Once implemented, the combined efforts of both have the power to become cultural artifacts because they can “enable, extend, or constrain human activity” (Remillard, 2011; p. 114). Acknowledging the existence of a powerful relationship between teachers who are designers and the curriculum being designed is therefore necessary because this participatory process is notably riddled with confounding factors that need to be unpacked, interpreted, and communicated (Remillard, 2005).

#### Frames for solving design problems

It is possible to explore and analyze the confounding factors that impact how a TDT navigates the challenges of developing a STEM-integrated curriculum by examining the nature of their conversations. Language is the primary means by which designers discuss, clarify, and develop their ideas as they attempt to identify the value of a proposed idea (Dorst, 2006). Once teachers are able to communicate the value of the artifact, they are creating, they can begin inductively working backwards via a “frame” (Dorst, 2011) that links the desired outcome to a set of yet to be determined working principles (e.g., “students will be collaboratively discussing real-world problems while searching for plausible solutions”). This process of “problem structuring” (Ertmer et al. 2008; Goel and Pirolli, 1992; Jeffries et al. 1981) or “framing” (Dorst, 2011) helps designers determine how a set of guiding principles might work in parallel with a yet to be conceptualized artifact. In order to reach an aspired goal or value, the artifact must be connected with a set of guiding principles (Dorst, 2011), which once combined represent the *solution* to the original problem. Tasking a TDT with the responsibility of pioneering and developing a STEM-integrated curriculum is particularly challenging because they must integrate a variety of related and potentially expansive conceptual ideas. In order to help a TDT take on this challenge, they must first determine what the desired endpoint could or should be.

#### Designing with the end in mind

Teachers as curriculum designers do more than just write lesson plans. Teachers essentially have a “voice” (Remillard, 2011) that speaks to anyone who uses the curriculum they create. Curriculum, once completed, is difficult to create because the output must function independently of the designer (Jonassen, 2000) meaning the author can no longer intervene or provide further guidance. The message a curriculum delivers must therefore be preconceived and provide a plausible solution to those who put it into action (Dorst, 2011). In regards to STEM-integrated curriculum, the value must reside within both localized needs and national standards (e.g., NGSS, 2013). Unfortunately, inexperienced designers struggle to first uncover a desired outcome or “value” (Dorst, 2011) because they tend to simultaneously generate multiple ideas in hope that a random collection of lesson plans will meet the aspired value (Hoogveld, Jochems, and Van Merriënboer, 2002). To counteract this, teachers must first be involved in conversations centered around a curriculum’s purpose, intent, and proposed value, which is precisely what Remillard (2005) conceptualized via her teacher-curriculum framework.

The theoretical framework constructed above identified two curriculum development “arenas” pertinent to the current study along with three conceptual lenses that could be used to analyze a TDT’s efforts as they create a STEM-integrated curriculum. The conceptual understandings revealed via this theoretical grounding when combined with the implications from the previously conducted literature review help clarify the study’s purpose and intent. Additionally, the frame justifies the study’s research design and research questions; the first of which was qualitative in nature and the later aimed at exploring the *processes* and *supports* that might enable successful STEM-integrated curriculum development by a TDT.

## Methods

### Professional development context

This study closely follows the actions and conversations of two TDTs during a large, federally funded STEM Education PD project (DUE-1238140). The project was developed in partnership with three large school districts in the Midwest (two urban and one suburban) with the overarching goal of helping teachers (grades 4–8) develop and implement engineering-integrated curricular units to facilitate the learning of major science concepts. During the PD, teachers participated in a three-week intensive summer PD program, where they first experienced STEM-integrated curriculum as learners and then collaborated in inter-district teams to develop a STEM-integrated curricular unit. Teacher teams then piloted the curriculum at a university-based summer camp with age-appropriate students. Teachers were supported by a STEM education graduate student throughout the project, including the forthcoming academic school year. During the academic year, each teacher implemented the curriculum in their respective classroom, working in partnership with their team to refine their curriculum for broad-scale dissemination and classroom implementation.

This study consists of data from the summer PD as teachers worked collaboratively in TDTs to develop STEM curriculum. Curricular units needed to include an engineering design challenge that fostered learning of a specific scientific concept(s) and also involve mathematical understanding of data analysis. Each unit was tailored for a grade level-specific audience. Prior to embarking on curriculum development, teachers engaged in professional learning about STEM integration aligned with the *Framework for Quality K-12 Engineering Education* (Moore et al. 2014). The framework was prominently featured and discussed throughout the entirety of the PD and was used as a guidepost for curriculum design and development. Additionally, during the second week of PD, teachers worked in content-specific groups (earth, life, or physical science) to explore more deeply the integration of engineering into a specific scientific domain.

Two additional supports were explicitly promoted within the PD structure. The first included the curriculum writing strategy known as *understanding by design* (Wiggins and McTighe, 1998, 2011). Understanding by design promotes a “backwards design” strategy wherein curriculum designers are prompted to unwrap content standards and to write a list of desired results and essential questions prior to designing assessments and subsequently lesson plans that align with the unit’s overall objectives. Second, a reflective partner or coach (York-Barr, Sommers, and Ghere, 2006) who was to utilize elements of instructional coaching (Knight, 2007) was placed with each TDT during curriculum design conversations.

### Research design

The participants from each of the TDTs in this study represent a single case (Merriam, 2009). That is, while each team generally operated independently of each other during the time of data collection, both teams were analyzed concurrently while searching for emergent themes and patterns. This applied case study demonstrates “an in-depth description and analysis of a bounded system” (Merriam, 2009; p. 43) that attempts to provide “thick descriptions” (Geertz, 1973) of two TDTs during a 12-day PD opportunity highlighted by elementary-focused, STEM-integrated curriculum design.

#### Participant selection

During the second summer of the project, 40 teachers participated, with 17 teachers choosing to focus their curriculum design within physical science, 11 within earth science, and 12 within life science. Data collection occurred within the physical science group because the physical sciences better support and connect with engineering and mathematics content standards (Guzey, Moore, and Harwell, 2016). Within the life science group, the opposite situation was uncovered, thereby excluding it from selection. The researcher’s own role and influence within the earth science group was considered potentially conflicting and therefore deemed an inappropriate fit for this study. From the potential pool of 17 teachers then, seven were “purposefully selected” (Patton, 2002) primarily based on graduate student-team pairings and past participant-researcher relationships. Table 1 provides further demographic information about each team. Six of the seven teachers selected had applicable experience developing and implementing a STEM-integrated curriculum just a year earlier.

**Table 1:** Team participants and demographic information

Team Engineering to the Rescue				Team Reckoning Force			
Name	Years	Position	School type	Name	Years	Position	School type
Matt^a^	3	Elementary	Urban	Derek^a^	5	Science specialist	Suburban
Nathan^a^	11	Elementary	Suburban	Michelle^a^	24	Science specialist	Suburban
Sammy	11	Science specialist	Urban	Evan^a^	26	Science specialist	Suburban
				Jill^a^	20	Elementary	Urban

^a^Denotes participation in previous year of project

### Teacher design team descriptions

#### Team reckoning force

Team *Reckoning Force* consisted of four teachers, three of whom worked in the same suburban district in different elementary schools as STEM specialists. The fourth teacher was in a gifted and talented elementary school in an urban setting. All four participated in the project the previous year. Team Reckoning Force was supported by Hank, a second year coach with 3 years of experience as a middle/high school physics teacher.

#### Team engineering to the rescue

Team *Engineering to the Rescue* consisted of three elementary teachers from the same district who taught at different schools. Two members of the team, Matt and Nathan, participated in the project the prior year. Sammy was new to the project but had participated in curriculum writing projects in the past. Nick, a second year coach and former mathematics/physics teacher supported the team.

### Data collection

The two primary sources of data collected were TDT conversations and individual participant interviews. Table 2 displays the stage during the PD when primary and secondary data sources were collected. Secondary data sources, used primarily for data triangulation and further contextual information, consisted of individual participant reflections, field notes, and curriculum development artifacts.

**Table 2:** Professional development overview and data collection timeline

Days	Activities	Data collected
1–2	-Project introduction -Engineering education framework introduced via make it better activities (engineering design focus) -Integration curriculum assessment discussed	-Individual participant reflections -Field notes -Audio and video of PD
Days		Data collected
3–5	-Science content groups split -Simple machines activity (bikes) -Force and motion activity (hover crafts) -Formation of teacher design teams -Introduction to understanding by design	-Individual participant Reflections -Field notes -Audio and video of PD
Days	Activities	Data collected
6–12	-“Open” curriculum development -Curriculum idea team share outs -Integration curriculum assessment check of curriculum	-Team curriculum design conversations^a^ -Participant interviews^a^ -Individual participant reflections -Field notes -Curriculum development artifacts

^a^Denotes a primary data source

TDT conversations were recorded via digital audio recorders during the final 7 days of the PD, which were primarily open for teams to utilize for curriculum design. Over 2300 min were recorded in total for both teams. Individual TDT members were each interviewed once for roughly 45 min towards the end of the PD using a semi-structured, open-ended, and responsive interview protocol (Seidman, 2013). Interview questions were reflective in nature and centered on the curriculum design process. Audio-recorded TDT conversations and participant interviews were digitally uploaded at the end of each day where they were later downloaded for analysis and transcription. Audio files ranged in length occasionally reaching over 200 min. Select turns of the audio were transcribed using Jefferson transcription conventions (Majors, 2007) to allow for a deeper reproduction and subsequent analysis of the raw data.. Researcher subjectivities and experiences facilitating curriculum design conversations determined which turns of TDT conversations were transcribed (Charmaz, 2006). Incidents not transcribed were time-stamped and summarized. Resultant transcriptions were finally inserted into a data management software program (NVivo Qualitative Data Analysis Software, 2014) for subsequent analysis.

### Data analysis

The inductive analytical strategies of this study align with those of constructed grounded theory (Charmaz, 2006). More specifically, data analysis began with a series of open/initial, incident-by-incident coding (Bazeley and Jackson, 2013; Glaser and Straus, 1967; Patton, 2002) of the study’s two primary data sources: TDT conversations and individual interviews.

#### Initial to focused codes

Transcribed excerpts from TDT conversations and participant interviews were tagged with short phrases and written descriptions during initial coding as transcripts were read and re-read. Secondary data (e.g., participant daily reflections) was also consulted during this phase to better contextualize and understand each participant’s experience. Coding at this time was intended to be “expansive” (Merriam, 2009) with the overall intent being to find patterns across the data corpus that could be housed within unique and exhaustive categories. A clustering strategy placed initial codes into broader, inter-related focused codes (Charmaz, 2006). Collated transcriptions could now be read and interpreted within a newly created conceptual category (Charmaz, 2006) in a manner similar to analytical coding (Merriam, 2009).

Each conceptual category now included disaggregated pieces of data that were pieced together via an analytical memo (Erickson, 1986). Analytical memos connected related, but distinctive pieces of data from the corpus in a narrative manner. At this point in the analytical process, secondary data sources were triangulated (Fielding and Fielding, 1986; Patton, 2002) and included within the analytical memo. For example, field notes from a whole group PD session were referenced to affirm TDT conversations dealing with the supports provided by a member of the PD team. The data corpus, now connected and categorized within one of five conceptual categories, could now be further examined. Table 3 contains an indicator description of each category along with an example extract. The five categories that emerged are expansive. Given TDTs discussed many topics, an attempt was made to create categories capable of classifying a large range of possibilities. With the data now packaged in a more easily understandable and approachable manner, the next stage of analysis was initiated.

**Table 3:** Cross-team focused codes, descriptor, and example extracts

Focused code	Indicator descriptor	Example extract
1. Team chemistry	Instances that indicated how a team generally operated or individual roles that were taken up. These could be deemed positive or inhibitory.	Jill: Now that you’ve presented this Evan, it sounds like we are in agreement that this is the way to go.
2. PD–supports/orchestrations	Mentions of PD activities or structures that were brought up in conversation or during an interview. Typically, these were brought up as being beneficial.	Derek: The whole point of us writing this curriculum is to share (it). That’s why we are doing this stuff.
3. STEM integration	Occasions wherein teachers discussed any of the disciplines of STEM education, STEM integration, or STEM curriculum. Engineering design challenges were prevalent here.	Nathan: As an engineering piece, what can they (students) build that will reinforce those learning targets.
4. Teacher ideas/influences	Occurrences where teachers brought ideas to the team or were influenced in their thinking about what their curriculum could be. Discussions of the idea generation process were also included here.	Samantha: We did not need to stay with this idea and feel like…let’s brainstorm some new ones that we like. Let’s be proactive because I’m not attached to this (idea) at all. I think at this point, I feel like I’ve had everything I have to say about it.
5. Facilitator role	At certain points in the process, graduate students (in a facilitative role) were deemed valuable to their respective teams. Influential instances were collated here.	Derek: We have been talking so broadly. I mean you’re shortening it to “How do we get non-bike riders riding?” it’s just a nice clean way to do that and it’s in student friendly language already. Hank: That’s kind of my goal.

#### Rational for selective coding

Advocating for a coding scheme to be used in a deductive manner requires a rational and justification. First, the borrowed categories must be compatible with the study’s purpose and theoretical frame (Ezzy, 2002; Merriam, 2009; Straus and Corbin, 1990). Next, previously created focused codes must connect with the proposed selective codes (Cohen, Manion, and Morrison, 2007). Finally, one must acknowledge how the selective coding scheme aligns with current theory and applicable research.

To begin, the purpose of the study was not to simply uncover *what* TDTs discussed during the curriculum design process (see Table 3). In order to analyze a data set containing lengthy, uninterrupted design conversations, it was necessary to first break down design conversations by topic (e.g., STEM integration) followed by a search for *the ways in which TDTs communicated their ideas* during the design process. This was necessary because language often times reveals a designer’s plans before an ideas is completely solidified (Dorst, 2006). Simply put, the five categories that emerged from focused coding allowed TDT conversations to be compartmentalized and contextualized, which could then be followed by selective coding. Ultimately, in order to better understand the collaborative process (Peercy et al. 2015) of STEM-integrated curriculum design and the nature of teachers’ design expertise (Huizinga, 2009, Huizinga et al. 2014) further analytical work was needed.

#### Selective coding process

Selective coding began by identifying the “characteristic” features teachers faced while creating a STEM-integrated curriculum. The following selective codes, derived from Jonassen’s (2000, 2011) broad-level characterizations of design problems, were therefore applied: (1) ambiguous specification of goals (2) no known solution path and (3) integration of multiple knowledge domains. By identifying each of these three characteristics, it became possible to discover the types of challenges teachers experienced when trying to design a STEM-integrated curriculum.

From here, a second layer of selective coding was applied to uncover *how* teachers attempted to work through a given predicament by using Goel and Pirolli’s (1992) “design problem invariants.” Each of the *invariants* identified by Goel and Pirolli’s (1992) focuses on an individual’s actions by identifying behaviors that involve either *problem structuring* or *evaluation*. For example, designers typically structure a problem in the beginning stages by compartmentalizing the problem into “modules” (i.e., modularity). Table 4 provides a more detailed description of the five most frequently coded invariants teachers employed during the curriculum design process. After analysis, it was discovered that TDTs spent a majority of their time in the preliminary stages of the design process, which limited the frequency of strategies often exhibited when the design process moves towards the latter stages (Goel and Pirolli, 1992). Dual layers of selective coding identified the *characteristics* of the problems teachers faced and the invariants, or strategies, they employed when tackling a given challenge.

**Table 4:** Selective coding schema for characteristics and invariants of a design problem

Selective code category	
Design problem characteristic	Indicator descriptor
1. Ambiguous specification of goals	-The goal for the set out task is lacking in specificity, it is ill-defined, vague, and left open for interpretation. Proper evaluation metrics cannot be used to determine how appropriate a given solution is for the problem in large part because of the uncertainty of what the goal truly is.
2. No known solution path	-Given the ambiguity of the goal, there is no definite set of actions a designer can or should take to reach that goal. There is no map to follow and essentially one must create the pathway prior to and while walking it, which in turn creates unfamiliarity and discomfort.
Design problem invariant	Indicator descriptor
1. Design problem structuring	-The starting point for designers lacks information and definitive goals; therefore, purposeful structuring of the problem needs to occur. This results in a set of specific actions as designers look for pertinent information and attempt to structure their task in a way that is comprehensible at the point in time that it was conceptualized.
2. Modularity/decomposability	-The complexity and size of a design problem inherently forces designers to compartmentalize it into “modules.” Because presupposed connections amongst the modules are contingent, designers will likely attend to some and ignore others. Also, as contingent connections invariably change, some modules are thereby ignored or lost all together.
3. Distinct problem-solving phases	-Designers will exhibit distinct behaviors and actions during each of the three phases of problem solving: preliminary, refinement, and detailed design. As a result, certain requirements are created that must be attended to during each phase, including but not limited to the amount of attention given to detail.
4. Incremental development of an artifact	-Provisional ideas that are discovered are incrementally nurtured by designers and rarely completely abandoned because there are no right or wrong answers to the problem. It is therefore permissible and acceptable to hold on to a given idea, which may eventually become a suitable artifact while it is steadily developed.
5. Personalized stopping rules and evaluation functions	-Evaluating any proposed idea or choice requires that designers make personalized evaluations because one can never know for sure what the “right” answer is. As a result, designers tend to institute experienced-based “rules” or strategies that allow them to move forward.

#### Empirical assertions and evidentiary warrant

Finally, a three-way contingency table was created that contained a frequency count of all previously described selective codes. This table was used to generate and investigate various empirical assertions. In total, 592 incidents were subjected to multiple layers of open and selective coding, of which 100 were deemed non-applicable during selective coding. Non-applicable extracts consisted of superfluous, tangentially related commentary about teaching or personal experiences. The three-way contingency table was used to generate and investigate a variety of empirically driven assertions. The count of incidents within a single cell of the contingency table ranged from just 1 to over 70. The three-way contingency table in and of itself was only used as an analytical tool and will not be featured in the results section (see Additional file 1).

Following Erickson’s (1986) guidelines for reporting fieldwork research, analysis ended by testing inductively generated empirical assertions. Proposed assertions were tested against the data corpus, while searching for confirming and disconfirming evidence. General assertions were constructed using the computer-generated, three-way contingency table (Fienberg, 1977). High frequency counts from the contingency table aided in identifying and examining potential assertions. For example, in order to reveal what TDTs did when they encountered a challenge without a simple or known solution, a particular cell of the contingency table could be identified and investigated to reveal how teachers responded to these scenarios (e.g., no known solution path and design problem structuring). Upon reading and re-reading these incidents and while exploring related pieces of data (e.g., curriculum design artifacts), initial assertions were tested against the data corpus. Assertions best supported by the evidence were considered representative of the data set as a whole. This synoptic reporting provided the study with a form of *general description* (Erickson, 1986). For ease of reading, specific description (i.e., representative transcriptions and interpretive commentary) will follow a single team. Again, the aim of this reporting is not one of proof; but rather as Erickson (1986) states to, “persuade the audience that an adequate evidentiary warrant exists for the assertions made, (and) that patterns of generalization within the data set are indeed as the researcher claims they are,” (p. 149).

## Results

The results are grouped into two sections, detailing the outcomes for two different TDTs. Before providing a more detailed, specific description of each theoretical assertion, an overview of the curriculum development pathway followed by each team is offered using a flow chart structure to guide the reader through the emergent temporal development of that team’s curriculum unit. Following this flow chart overview, a synopsis unpacks the flow chart elements and details how the ultimate assertion emerged via investigation and analysis of the aforementioned contingency table. Each section then ends with an empirical assertion that incorporates relevant theoretical perspectives.

### Remaining on familiar ground

**Fig. 1:**
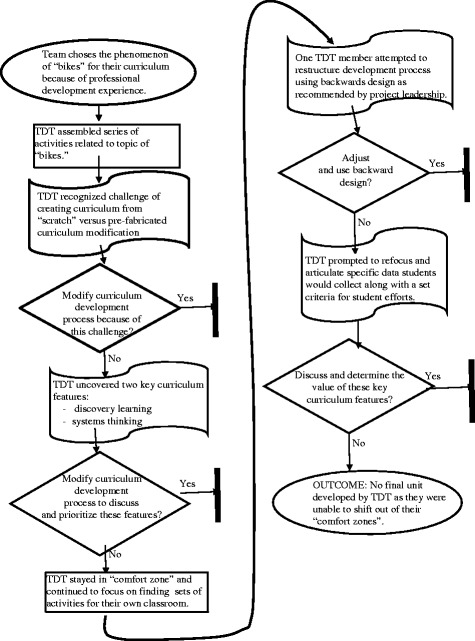
Map of team Reckoning Force’s approach to curriculum unit design

Both TDTs encountered a challenge with minimally defined success criteria (Jonassen, 2011). An examination of a related group of cells within the contingency table revealed how the overarching ambiguity of the design problem made it necessary for both teams to structure the problem in order to move forward (Ertmer et al. 2008). High frequency counts were noted in a group of cells in the contingency table (*design problem structuring*, *modularity/decomposability*, *distinct problem solving phases*), which prompted further investigation. Briefly put, an examination of both sets of TDT conversations revealed how the teams’ choices influenced the respective pathway they each followed. Team Reckoning Force will be used to describe the study’s first empirical assertion because the design decisions they enacted ultimately prevented them from moving beyond the initial stages of the design process. The development process employed by Team Reckoning Force is outlined in Fig. 1.

#### Team reckoning force summary

Team Reckoning Force wanted to create a STEM-integrated unit that utilized a variety of science, engineering, and mathematics standards using bikes. The team wanted a unit that asked students to modify one or more aspects of a bike to encourage usage by atypical riders because a new design met a specific group’s needs (e.g., disabled veterans). The potential benefits of designing a STEM-integrated unit involving bikes intrigued the team after they experienced a similar activity during the PD. The team assumed they could build from this experience and compile a variety of lessons and activities for their unit without engaging in extended conversations about how the unit would function. The assembled sequence of activities they developed contained the preliminary details of a variety of lessons they felt would best be developed by individual “camps” to “fit” their contrasting classroom contexts.

Team Reckoning Force needed to employ a variety of strategies they typically do not use every day in the classroom. The main supports put in place to help the TDT navigate the barriers of STEM-integrated curriculum development failed to offset the team’s usual process for finding and modifying lesson ideas for classroom use. The team never developed a shared vision of the unit’s overall purpose so it was difficult for them to see how a proposed idea and/or lesson could work in their classroom. Opportunities to get the team on the same page were limited but when presented would have likely required team members to divulge and discuss their own beliefs about the value of integrated learning experiences (Remillard, 2011).

#### Finding a starting point

As previously mentioned, team Reckoning Force took up the main focus of their unit from one of the whole group PD activities.

Derek: You know, I think probably our unit plan…one of the major things is that our unit plan is coming off of one of the lessons that we did with the physical science group. That was a big “aha” moment for us, to have something that we thought our unit could be about.

As has been reported elsewhere (Koh, Chai, Wong, and Hong, 2015), the team latched on to the idea and incrementally developed it throughout the curriculum design process. The team wanted a starting point and discernible context for their unit. They also viewed bikes as an “engaging topic” for their elementary-aged students who frequently rode bikes. The learning environment experienced by the team during the PD involving bikes was familiar and therefore a natural choice as the unit’s central focus.

Team Reckoning Force; however, struggled to move much further beyond this starting point. The hidden complexities of bikes and the variety of science standards that could align with a unit focused on bikes made the next steps for the team challenging. Evan, a veteran teacher of 26 years, discussed how challenging it was to be a curriculum designer, in comparison to being a classroom teacher during his individual interview.

Evan: Because you’re taking…you’re going from scratch, yeah. A lot of the times, if you have your classroom and you're working with a curriculum, well there’s a lot of assumptions that are pre-built and a lot of the directions are pre-built and you can take it and spin it and make it your own. But this comes from…there’s nothing before you start.

As Evan noted, the “assumptions” or working principles of a curriculum need to be discussed and determined during the curriculum design process because they are not provided or easily recognizable. In the past, Evan was able to “spin” the propositions provided by a pre-fabricated curriculum. As a curriculum designer, however, he now had to instill these propositions from “scratch” within the lessons the TDT needed to write. In other words, he no longer had to “make it his own,” but rather needed to make it himself. Evan and the team needed to employ a different set of skills.

#### Remaining in the initial stages

During a team conversation, Derek described a few pieces of the problem they were attempting to solve.

Derek: Well…I think the way we’ve been talking, we want there to be this discovery piece in there. Um, we want them to be able to talk about the systems in a bike and all the pieces; and how they move together and how they move separate. (We want them to know) how each piece (of the bike) works. So I think a big part early on is maybe…I mean part of that, I don’t know…do we jump into background science right away or do we jump into how a bike works right away?

In this early declaration to the team, Derek identified two key preliminary features of the curriculum as he personally envisioned it. He felt that a STEM-integrated curriculum should contain elements of “student self-discovery” and that curiosity would naturally drive students towards discovering how bikes work and how they could be improved. He was also interested in having students learn about the science of bikes, particularly as it related to bikes being an interconnected “system.” From here, he doubted his initial instinct the next decision the team needed to make was whether or not to front load the science content surrounding bikes (e.g., balance and motion) or “jump” into the logistics of bikes. Derek did, however, identify two potentially useful guiding principles that could have directed their curriculum in a specific direction (Dorst, 2011). Each of these guiding principles (i.e., “systems thinking” and “discovery learning”) would have ultimately influenced the team and the lessons embedded in the unit.

**Fig. 2:**
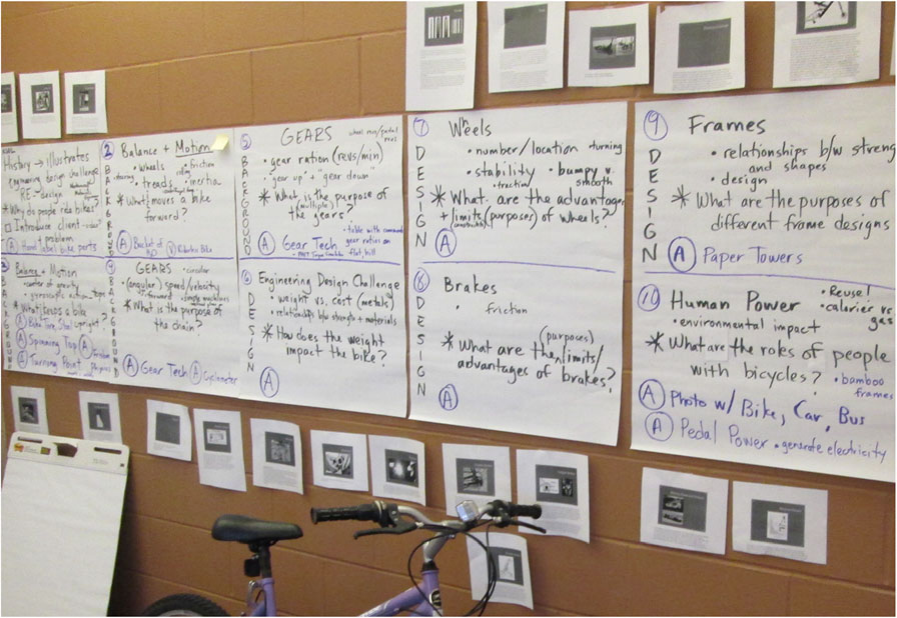
Teacher design team curricullum development work area

Derek nevertheless jumped from these two propositions and began thinking about the lesson sequence to begin the unit rather than incrementally developing the details of each proposed curriculum feature. Figure 2 displays how the team organized their ideas. They decided to surround their work area with large posters that outlined their lesson sequence. Each poster contained the inklings of a lesson: *general topic* (e.g., “wheels”), *guiding question* (“what are the purposes of different frame designs?”), *objectives* (e.g., “the relationship between strength and shape”), and *necessary resources* (e.g., “cyclometer”). The team eventually identified an initial lesson denoted as “background: balance and motion.”

The team ultimately determined they could begin drafting up lesson plans by referencing the starting points and ideas listed on these sheets of paper. In a sense, the team behaved as teachers typically do in the design arena (Remillard, 1999) by drafting lesson plans for implementation in their respective classrooms. The team’s natural tendency was not to map out final student assessments or unit learning goals, key features of *backwards design*, but rather to draft lesson plans. The team continually revisited and referenced the posters shown in Fig. 2. Unfortunately, proclaiming bikes as the central context of their unit and creating these posters complicated matters for the team. With the science of bikes identified early on as the unquestioned focus of the unit, the team spent the remainder of their time together trying to work through numerous issues, primarily by finding and adapting lesson ideas that might work for their individual classrooms (Debarger, et al. 2017), not necessarily as a part of the team’s unit.

The five pieces of large poster paper in Fig. 2 represented the teams only written endeavor. From here, they wanted to move forward by writing loosely related lesson plans that “worked best for them” in their given contexts (STEM specialists and an elementary generalist), which was difficult to do. Michelle noted during her individual interview near the end of the PD how the team utilized the posters described in Fig. 2.

Michelle: I think as we have this up (the posters)…everyone can, as were talking…Everyone’s kind of looking and figuring out what’s going to work best for them.

In the end, the team determined the next best step was to create lesson plans based on the lesson sequence they co-created via these posters for their respective classrooms.

#### Resorting back to a familiar, comfortable pathway

At this point, the team knew their time together was ending and that they would soon need to implement a pilot of their curriculum for a group of students attending a summer camp. Their concerns still dealt with their individual classrooms and the lessons they would eventually need to implement in their own classrooms during the upcoming school year.

Evan: I think we just have a different context. You know our contexts are a little different so it’s worth (discussing) how we’re going about this a little different. I think we can get it to go together and can still make it work for both (groups) of us you know. There’s no judgment like that at all, but I think the way…some of the needs we have for the way we need to do the work is a little different.

Derek: Yep.

Jill: But I think were still hinging on the same thing which is…it’s this…[the posters]

Evan: Right.

The team ultimately felt they were made up of two different camps and that a STEM-integrated curriculum involving bikes could not be devised to meet their unique needs. Evan suggested the team discuss their different contexts in an attempt to get the unit “to go together,” also noting this could be done without “judgment.” It was however deemed too difficult to view the unit holistically as a system composed of individual lessons or parts (Reigeluth and Avers, 1997) that could work in numerous classrooms so the team moved forward with a new agenda.

The team failed to move beyond this predicament. Shortly after the above discussion, Jill declared, “whatever we write will be what you guys are doing and if I do it differently, that'll be just like teacher enrichment notes.” Upon referencing other exchanges in the data, it was uncovered this strategy was denoted as resorting back to your “comfort zone.” In the end, team Reckoning Force did not move beyond the ideas they initially developed on the posters and never wrote an initial draft of their unit to share with the project team.

#### Providing curriculum design support

Team Reckoning Force needed their initial curriculum ideas translated from the sheets of poster paper in order to move forward. To help the team make this translation, a facilitator with experience developing STEM-integrated curriculum and a series of “backwards planning” templates (Wiggins and McTighe, 2011) were provided. The templates consisted of three stages and prompted the team to first “unwrap” a series of standards in order to define a tangible end point (i.e., *desired results*), which could be further defined by writing a *big idea* and *essential question* for the unit. In order to do this, appropriate standards needed to first be identified and unwrapped.

The team however preferred to engage in conversations about previously used lessons from their own classrooms they could modify to fit into their yet to be fashioned unit (Prawat, 1993). Hank, the graduate student paired with the team, realized the teachers’ intuitions were taking over and attempted to bring their attention back to the promoted method for creating curricular units. Below, the team needed to determine if their students could successfully relate a lesson about the revolutions of a bike’s tires and distance traveled to the overall design of a bike. Hank, after listening to the conversation, responded by bringing the team’s attention back to the standards that had yet to be identified.

Hank: I’m thinking if that’s the real guts of this activity that hopefully we’re going to be able to design the activity to support data collection and analysis, right? I guess I’m just asking, like what’s the meat of this activity? What are students doing that you say ‘ah ha’ some science and math is happening here? What do you see your students being able to accomplish with this activity? From here, we will unwrap and design this activity around that goal. Is that clear as mud? I don’t know if I’m making any sense at all (laughs).

Hank wanted to create a bridge between the teachers’ intuition to remain in the design arena and the mapping arena by appealing directly to their prospective students. He emphasized student activity (e.g., “What are students doing…”) and backwards planning (e.g., “we will unwrap…”), but in the end indicated he was uncertain if his questions or suggestions were useful (Binkhorst, et al. 2015). During his individual interview, Hank stated he needed to balance the tensions (Becuwe et al. 2015) he experienced between his team’s preferred method for developing curriculum and the project-sanctioned process for developing STEM-integrated units. The team understood the purpose of the backwards design templates, but did not use them. Backwards design intended to first lead the team towards determining a desired endpoint, which would be followed by discussing the best ways to reach that destination. Ideally, teachers would expand their thinking during this process while developing a more focused understanding of the unit’s overall purpose (Wiggins and McTighe, 2011).

However, the team had not yet articulated their own thoughts and feelings about the value of STEM-integrated learning experiences, which made it challenging for them to enter into this space (Dorst, 2011; Koh, Chai, Wong, and Hong, 2015). In this example, as was common in others, the team was not encouraged to engage in conversations about the potential value of the curriculum they were designing. Instead, they were directed to break down hypothetical classroom activities (e.g., a data collection/analysis activity involving tires) in order to identify plausible content standards that could be unwrapped to determine desired student outcomes (Wiggins and McTighe, 2011). Other conversations, in addition to the one presented above, also demonstrated how the team favored finding and modifying lessons rather than embracing a “backwards design” planning approach. In the end, the team wanted to remain in their “comfort zone,” which clearly was the design arena (Remillard, 1999).

#### Clearing ambiguity with conversation

This final exchange highlighted how Evan, the veteran of the group, inadvertently suggested a different pathway for the team to pursue. The team struggled to make actionable curriculum design decisions and to move forward during the design process largely because they continually attempted to design lesson plans they could “fit” into each other’s respective classroom. The below proposition to the team, as a contrasting piece of evidence to the data above, represented a different pathway than the one the team generally followed.

Evan: Part of the challenge here is as they [students] create the design is to somehow have a prototype that they can do analysis around and gather data that is calculable and observable. This can then inform redesign. That’s part of the engineering design process; that loop of redesign. Real engineers tend to redesign, redesign, redesign and do many different derivations of the same idea before they're done. For our limited experience with students (in the classroom) we have a loop where we do one design and then do some analysis of the performance of that (student-created) design. This is done around the parameters and criteria (we’ve set). After students have generated that data, they make this [the design] better with changes.

Evan tended to focus his conversations on his students and the experiences he would eventually facilitate with them “sitting around the table top.” Here, he attempted to connect the realities of the classroom with the world of “real engineers”; a worthwhile conversation to have. He cited two constraints he typically faced in the classroom: (a) having tangible prototypes that could yield reliable data, along with (b) classroom time restrictions. He followed by suggesting a plausible next step for the unit’s engineering design challenge. He exhibited his understanding of the engineering design process and identified two specific abstractions he felt needed to be resolved via active discussion (Dorst, 2006): (1) determining what data students would be generating, collecting, and analyzing, followed by (2) the need to define and determine a series of “parameters and criteria.” His practitioner-based, insightful view of engineering and STEM education were underscored here (Boschman, McKenney, and Voogt, 2014).

Underlying Evan’s ideas were a set of guiding principles that disclosed how he felt engineering should be integrated into elementary science classrooms along with his views of how students should be engaged in learning experiences highlighted by engineering design (NRC, 1996). Further discussion, development, critique, and evaluation (Molenda, 2003) of his call for action unfortunately never occurred. It has been reported elsewhere that analysis activities are often overlooked by teachers (Hoogveld et al. 2002; Huizinga et al. 2014), which was reaffirmed here. Had the team pursued Evan’s propositions, they likely would have further developed their design expertise, particularly their formative and summative evaluation skills (Huizinga, 2009). Evan laid out a pathway for the team by recommending full engagement and active discussion of the learning experiences he envisioned would be embedded within *their* curriculum (e.g., “student-center design”), which also never occurred.

Again, this example was included as a contrasting piece of evidence to the manner in which the team tended to operate. Proclamations like this were rare and not followed by extensive inquiries into the propositions put forth. Instead, design conversations remained geared around what types of experiences students should be engaged in and not how students learn best during integrated learning experiences (Dorst, 2011).

### Theoretical considerations and first empirical assertion

Team Reckoning Force continually exhibited behaviors during their time together similar to a group of teachers preparing for upcoming classroom instruction. The team stuck with their initial design idea (Hoogveld, Jochems, and Van Merriënboer, 2002) and moved forward by individually developing a sequence of lesson plans with minimal consideration of how the lessons would fit together as a whole (Reigeluth and Avers, 1997). The lack of clarity surrounding the unit’s overall purpose inhibited the team from visualizing how it could be implemented in classrooms other than their own, which ultimately split the team in two. Because the team remained in the design arena (Remillard, 1999), they seemed to expect an “aha moment” to take place wherein a series of lessons and activities would suddenly be discovered that could easily be transplanted into the unit and work in everyone’s unique contexts.

Instead of discovering a “voice” (Remillard, 2011) to unify the team’s thinking, they determined each member could figure out the message the curriculum would deliver retrospectively. The resultant theoretical assertion was derived via analysis of team Reckoning Force’s collaborative curriculum design efforts:

When a TDT is not prompted and/or enabled to first lay out and articulate the overall value (Dorst, 2011) of a STEM-integrated curriculum, they will *rightly* remain within and therefore operate as a team of teachers within the *design arena* (Remillard, 1999).

It was difficult for the team during their time together to find common ground and create a STEM-integrated unit for an outside audience. Instead of formulating a unified understanding of *how* STEM-integrated curriculum could work in anyone’s elementary classroom, the team remained within their accustomed “comfort zones” and continued searching for lesson ideas and thinking of ways previously implemented lesson plans might be spun into their “new” unit.

### Personalizing the curriculum design process

The following section describes how team Engineering to the Rescue made personalized decisions during the curriculum design process. The team wanted an authentic engineering design challenge students would “get something out of” while applying their understanding of force and motion (NRC, 1996) as student built a rescue vehicle that could traverse multiple surfaces (e.g., ice, water, and sand). Student-created rescue vehicles were intended to represent a “realistic analogue” or prototype for an engineering firm to be utilized by a local fire department.

**Fig. 3:**
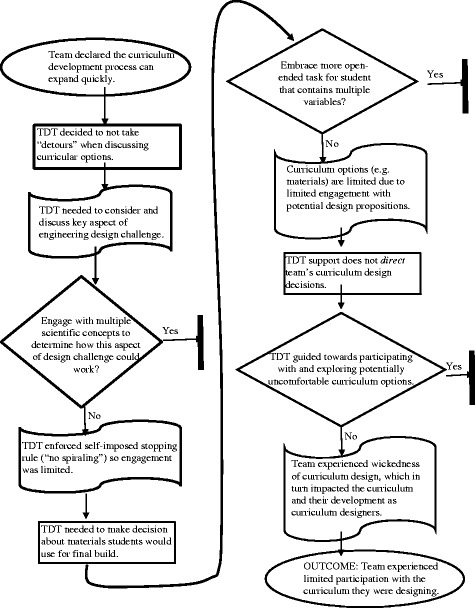
Map of team Engineering to the Rescue’s approach to curriculum unit design

Team Engineering to the Rescue discovered there was no definitive pathway to follow while creating a STEM-integrated unit and therefore made design decisions by formulating *personalized evaluations* (Goel and Pirolli, 1992) of prospective curriculum design options. The frequency of these occurrences (63) from a single cell on the contingency table (*personalized stopping rules/evaluation functions*, and *no known solution path*) stimulated further exploration of the underlying pieces of data. An assertion emerged that described how the team used “personalized stopping rules” (Goel and Pirolli, 1992) to navigate the curriculum design process. This unfortunately prevented them from fully engaging with the teacher-curriculum *participatory relationship* (Remillard, 2005). The team’s past experiences designing engineering-focused curriculum were identified as an influential factor that limited how the team engaged with the curriculum. The curriculum unit development process employed by team Engineering to the Rescue is outlined in Fig. 3.

#### Team engineering to the rescue summary

Team Engineering to the Rescue participated with the curriculum they were designing in a limited manner. As a result of two member’s past experiences with engineering-focused curriculum, they prevented themselves from going further down various pathways they uncovered. They consistently reinforced a stopping rule aimed to keep superfluous content standards, scientific concepts, and lesson ideas from entering the curriculum. This decision dictated how the team operated and altered the curriculum they were creating. In sum, curricular options were limited and/or simplified. The type of team-based discussion and discourse one might expect to unfold while searching for the solution to a design problem (Dorst, 2006) did not emerge because the team typically did not investigate an idea’s merit if it meant additional, albeit applicable, conceptual ideas were embedded within the proposition.

#### The past’s influence on the present

Team Engineering to the Rescue needed to have unique and meaningful conversations about the curriculum they were designing in order to be successful. Sammy felt she talked a lot more than the others, noting that as a group, “there’s been no conflict,” but at times she needed to “figure out when I should be quiet.” Because of Matt and Nathan’s experiences the year before, they influenced the team’s decision-making process (Huizinga 2009). Sammy, despite having previous experiences developing curriculum, was not a dominant figure and therefore minimally influenced how the team functioned. Below, Matt detailed how this happened.

Matt: The whole process of how we went through it [curriculum development and implementation] last year and how what we planned (to do) actually played out in the classroom. I think that’s kind of helped us…to keep it [the unit] more focused. Nathan has actually had the same experience too. He actually said something similar where last year his unit kind of just kept expanding and expanding and got too big.

Matt referenced how the curriculum he designed and implemented last year indicated to him that a STEM-integrated curriculum needs to be “focused.” Both Nathan and Matt learned curriculum can “expand” quickly, and this can be problematic. Below, Matt and Nathan further described their system for designing curriculum this year.

Nathan: What we learned…I think what I learned from last year is that you can come up with the concepts and it can all of a sudden grow into some huge monster. I think that was our problem last year.

Nick: The question is, are you using this monster in a good way?

Matt: I agree with that 100 percent!

Nathan: In a bad way because it seemed like it [the unit] just all of a sudden developed into this…it just kept expanding, expanding, and expanding.

Matt: Yeah, I agree.

Nathan: And I think you need to keep it focused, just very simplistic. Being able to just hit those things [standards] without having all these other detours.

Sammy: You said that too many benchmarks…you were trying to do too much?

Matt: Yeah I think it’s exactly what Nathan said…we start out with one idea and then it kind of kept spiraling upward…almost just growing and growing. I think there were a few (standards) we just kind of threw in there without hitting really well.

Nathan: Yeah.

Nathan and Matt detailed how they felt STEM-integrated curriculum should be designed. The integrated nature of the final product naturally brings with it ideas from multiple disciplines, which as Matt discovered, resulted in some standards not being adequately “hit” the year before. Nathan justifiably did not want to deal with a “huge monster” again this year so he and Matt both decided to keep things “very simplistic.” In many ways, this strategy represented a form of problem structuring (Goel and Pirolli, 1992). The team was using pertinent information drawn from personal experience to make their task more comprehensible because it was so complex. The team continually used this personalized stopping rule to make decisions. When someone was “spiraling,” team members quickly jumped in to stop themselves or others. Spiraling occurred when too many ideas, content standards, or activities were being considered all at once. The team wanted to compartmentalize their task into “modules” (Goel and Pirolli, 1992) in a sense, so they could focus on key elements of the unit and avoid “detours.” Had the team understood these detours were a necessity of STEM-integrated curriculum design, they may have embraced this discomfort (Remillard, 2005) and further explored multiple knowledge domains (Jonassen, 2000) with less reluctance. It is not to say that discovery of these connections is easy, but at least it is a possibility with integration of the disciplines now being actively promoted and pursued.

#### Stopping rules at potential turning points

Towards the end of the PD, the team discussed how “background or challenge activities” should be used to support students prior to the more open-ended design challenge; a strategy beginning to be advocated for (Guzey, Moore, and Harwell, 2016). In this instance, Sammy proposed a potential background activity dealing with mass. About 1 min prior to the below incident, someone noted it would be, “a good goal for us today…to fill in a little bit more of the backwards planning template…to revisit that,” which the team acknowledged and noted would be completed later in the day.

Sammy: I was thinking that they [students] would have to pick up a load and put that on (their vehicle). How does the mass change it as a challenge activity? But what I don’t want is for it to spiral. So I think if we go back to the UbD [understanding by design] process and do that first…and then make a criteria chart or whatever it's called.

Sammy suggested students grapple with an important variable, mass, while designing and building their rescue vehicles. Originally, the team considered using an apparatus to lift a “load,” but this idea was scrapped because it was deemed too complicated for students to construct a lifting mechanism *and* a vehicle. In this example, Sammy rightly wanted to discuss and assess the benefit of developing a sequence of lessons that would provide students with an understanding of how mass impacts force, motion, and friction (Huizinga 2009). Instead of starting this discussion, however, she quickly stopped herself from “spiraling” and decided a “criteria chart” could be created to address the issue. The team never constructed a criteria chart and instead chose to draw on a resource Nathan previously used in his classroom (Remillard, 2005); ultimately selecting an activity he called “spring scale sleds” instead.

Because the team had predetermined the content standards their unit would be aligned with, they limited discussion of the multiple, inter-related scientific concepts (e.g., friction and force) that could have been incorporated. Therefore, instead of contemplating Sammy’s suggestion and further discussing the applicability of Nathan’s activity, they moved on and presumed it would work without extensive analysis (Huizinga 2009). During his individual interview, Nathan elaborated further on how the team reinforced this “stopping rule.”

Nathan: I know that Sammy has had a couple of times where (she would say) it would be really cool if we could do this…and (I would say) yep that sounds cool, but let's just try to make sure that we….we just really need to stay focused on these key elements because (we only have) the ten days (in the classroom).

Nathan understood multiple ideas get mentioned while designing curriculum, but in the end, most fail to make it into the curriculum that gets enacted. The team set the unit’s timeframe at 10 days and knew if they entertained too many ideas during their time together they might not accomplish what they set out to do. The team’s “no spiraling” rule was not only enforced to help them stay on track but it also limited how deeply they dove into a topic particularly when it involved integrated conceptual ideas.

Early on, the team remained more open to and investigated multiple, “overarching” curriculum ideas. (e.g., storing water during a trip in outer space, building a wheel chair, and creating a wind-powered vehicle). However, once they landed on “rescue vehicles” as the unit’s focal point, they struggled to unpack and evaluate any supplementary design ideas the unit would contain (Hoogveld, Jochems, and Van Merriënboer, 2002). It was wrongly assumed the team would naturally engage in collaborative conversations as problems arose, which would prompt the team to refine and define a unified, practitioner-based vision of elementary-based, STEM-integrated learning (Boschman, McKenney, and Voogt, 2014). The team received some direction and guidance (e.g., “backwards planning”), but likely not enough thereby causing them to be lost at times (Pintó, 2005). The team used a “pros and cons” strategy to make decisions, but this alone did not fully address the challenges with idea analysis they encountered (Huizinga, 2009) The team’s personalized stopping rule when put into action tended to limit curriculum options rather than drive the team’s thinking towards new possibilities.

#### Limiting the curriculum based on stopping rules

The success of the team’s unit rested on a culminating engineering design challenge wherein students would build a model “rescue vehicle” that needed to travel over varying terrains during hypothetical rescue missions. Below, Sammy described where she thought the starting point for this open-ended task should be.

Sammy: You [Nathan] said earlier to think about the standards and I think that makes me lean towards having a base vehicle that we give them [the students] to start with. It’s not mechanical engineering so if we give them a base vehicle they can modify it. I think it [the task] will be more (focused) on the standard.

Sammy referred back to Nathan’s earlier request that the unit’s standards remain constant. Consequently, Sammy determined student’s autonomy should be limited during the design and construction of their model vehicle because expanding the task would eventually impact which content standards would be included in the unit. Students therefore would get a pre-fabricated “base vehicle” upon which a few modifications could be made (e.g., wheel type) as opposed to giving them the materials to build a vehicle from scratch. Again, just as Sammy previously decided not to delve into a team discussion about the ways in which mass impacts the friction exerted on a moving object; she elected here not to contemplate the complexity of the build and design challenge that the unit would end with.

From here and with a starting point decided (i.e., base vehicle), the team began to discuss what types of “wheels” (e.g., tires and treads) students would have available for their vehicles; a key aspect of students’ designs. Here again, the multitude of options the team could have considered was limited by the team’s stopping rule. Matt was charged with determining material options because he enjoyed tinkering with the materials students would eventually use. On the team’s previously mentioned “pros and cons” document, he noted that asking students to design and build a fully functioning rescue vehicle would be “materials and time intensive.” The team pressed on regardless and now needed to make an important decision about the materials that would accompany their curriculum.

Matt: It might make sense to have like…um…maybe like one or two options for just the…I don’t want to give them too many choices for the car bases because the difference there would be more wheels versus treads.

Nick: Right.

Matt: Maybe (we have) just a couple of different choices (for students) to choose from; just not to overwhelm them.

The team wanted the context and appeal of their unit to mirror that of the real world (NRC, 1996), therefore the vehicles students created needed to traverse the types of terrains rescue vehicles might encounter while saving someone in varying environments (e.g., snow, water, and sand). Rescue vehicles undoubtedly utilize a variety of mechanisms to accomplish this task, which in turn impacts how well they perform on any given terrain. Selecting a specific wheel type would impact the force needed to change a vehicle’s direction and influence the amount of friction created due to a tire’s design. Matt’s localized knowledge of a potential classroom constraint (Davis et al. 2011) and decision to give students just two options may have been warranted given the amount of time students would need to not only select but also to perform the analysis necessary to determine which type of tire works best on a given terrain. The main takeaway here is the team again limited an aspect of their curriculum because they “stopped” themselves from digging deeper into the issue.

Remaining open to and discussing the impacts of giving students multiple tire options, while seemingly trivial, would have likely required further structuring of the problem they encountered and forced them to ask and find answers to new, unanticipated questions (Ertmer et al. 2008; Jeffries et al. 1981). Answers to these questions would likely have been found by referencing the relevant standards and seeking out new sources of information that would need to be read, interpreted (Remillard, 2005), and reported back to the team (Dorst, 2006). From here, the team could make an informed decision about wheel types by balancing the affordances and constraints (Edelson, 2001) of the proposition.

On a related manner, it is worth reporting how Matt embraced his role as the “materials guy.” His active involvement with this key aspect of the unit positioned him as a resident expert amongst the team, which in turn limited his teammates expertise on the manner and resulted in superficial conversations between him and the rest of the team (e.g., “How’s the build going?”). This unbalanced understanding of the curriculum between team members limited some individual’s engagement and interaction with the curriculum, which when coupled with their decision to not spiral, hindered the entire team’s ability to fully participate with one another and the curriculum ideas they were considering.

#### Supporting the teachers and the curriculum

To end this section, recall team Engineering to the Rescue also included Nick, an experienced curriculum designer. Nick’s role on the team was unclear at times, and his suggestions were occasionally perceived as evaluative, which he recognized.

Nick: Part of it is juggling the balance between me wanting to talk my ideas out…because that’s how I need to process (them), but then also knowing that if I’m stating my ideas out loud it’s having an influence (on the team) which maybe more of an influence than I want to have.

Nick preferred to process his thoughts verbally and felt at times when he did this it influenced the team too much. He therefore hesitated to intervene if he felt it would impact the team’s general way of operating. This decision may have stemmed from Nick’s understanding teachers prefer a certain level of control over the decisions that impact their classrooms (Little, 1990). In addition to the response above, he also called the team “self-directed” and noted his role was to keep everyone “moving forward.” Despite Nick’s efforts to just remain a member of the team and not an evaluator, the teachers still looked to him for help determining the worth and value of their design ideas. Nick’s decision to limit his “influence” over the team positioned him as a partner that provided evaluative comments when beckoned, but not necessarily as a partner who could help the team expand their thinking. This predicament is further described below.

There is an intricate balance, or “juggling,” at play here with Nick and the team. While engaged in curriculum design, the team experienced many opportunities to make decisions and in turn learn from the consequences of those decisions. An experienced facilitator *might* be able to prevent detrimental decisions from being made if recognized in the moment. However, when facilitation becomes about preventing “mistakes,” it undoubtedly impacts a team’s ability to grow as curriculum designers and develop their personal design expertise.

It is therefore critical to note the type of support given to a TDT will impact both how the team progresses as curriculum designers (Becuwe et al. 2015) as well as the resultant curriculum. There needs to be a balanced approach when supporting a TDT because the nature of and extent to which curriculum development supports are provided may have potentially conflicting impacts on both the designed curriculum and the TDT. As exhibited here, team Engineering to the Rescue experienced the wickedness (Jonassen, 2000) and full spectrum of complications that arise when designing a STEM-integrated curriculum without continually being told how to proceed. This in turn influenced the team’s understanding of how to develop integrated curricular resources as well as the curriculum they created. And while it is not possible to know with certainty what might have happened if they continued to develop and discuss the curriculum ideas they continually prevented themselves for diving into, one cannot question Nick’s decision to let the team follow the pathway they did because facilitators should not necessarily be viewed as curriculum design experts either (Becuwe et al. 2015).

### Theoretical considerations and second empirical assertion

Team Engineering to the Rescue continually limited the extent to which they would expound upon an idea throughout the curriculum design process because they inherently knew they themselves, and eventually others, would need to take up the propositions embedded within their curriculum and create an integrated learning environment highlighted by engineering design. At the time, it seemed appropriate and necessary to prevent themselves from further engaging in uncomfortable and complicated endeavors, which in turn simplified the curriculum they developed. Communicating the message of a given curriculum along with a process for reaching the vision of that message is not an easy task so it seemed essential to keep things simple. Again, certain responsibilities and pressures accompany the curriculum development process because curriculum resources, as artifacts or tools, “are part of the material world made and used by humans to accomplish goal-directed activity” (Remillard, 2005; p. 114). A teachers’ perception of their own capabilities in the classroom along with others who may implement the curriculum therefore influences the lesson plans that ultimately get developed.

Seven of the eight teachers in the study previously experienced how curriculum resources dictated activity in the classroom the year before. Given the curriculum being created this year would again be widely disseminated, it is possible that both teams realized their individual interpretations of integrated learning experiences could influence someone else’s perceptions and enactment of STEM integration in the classroom. Writing integrated curriculum capable of communicating a coherent vision is complicated, and the team therefore invoked a stopping rule to make the task more manageable. Herein lies the crux of involving practicing teachers in the curriculum development process. They will inevitably apply familiar strategies and shortcuts in order to accomplish the task in the time allotted even if it is not how “experts” would do it (Kerr, 1981; Nieveen and Van der Hoeven, 2011).

Remillard (2005) stated both teachers and the text of a curriculum, “are engaged in a dynamic interrelationship” (p. 221), because the ideas from one source, once interpreted, have repercussions in the classroom when implemented. This realization, while currently recognized only with pre-fabricated curriculum, has not yet been acknowledged or recognized when curriculum is still under development. Curricular resources still under development and therefore not yet fully conceptualized still contain the preliminary vision of what will eventually unfold in the classroom and therefore still *participates* with the teachers involved in the curriculum development process. In other words, Remillard’s (2005) “participatory relationship” likely perseveres. The impacts of this relationship on classroom teachers who are also asked to be curriculum designers represent the study’s second empirical assertion.

Involving practicing teachers in the curriculum design process requires “participation” with the curriculum ideas they are contemplating (Remillard, 2005) because in the end, the curriculum’s resultant lessons will be taught in their own and other’s classrooms.

Remillard (2005) initially conceptualized the teacher-curriculum “participatory relationship” occurring only with pre-fabricated curriculum; the evidence presented here suggests it remained influential with teachers during the curriculum development process as well.

## Conclusions

### Issues with the preferred arena of collaborative curriculum design

In regards to the study’s first research question concerning the *processes of collaborative curriculum design*, the importance of helping teachers “break free” from comfortable curriculum development habits cannot be underscored. As evidenced in the results, both TDTs held preference for the design arena as opposed to the mapping arena (Remillard, 1999). Within this particular arena participating teachers struggled to utilize their localized knowledge in innovative ways for more generalizable use (Boschman, McKenney, and Voogt, 2014). Both TDTs were experienced, spent ample time together, had a variety of supports, and participated in activities during the PD representative of what STEM-integrated instruction could look like; yet it was difficult to move out of the design arena. In the end, neither TDT engaged in the process differently (Prawat, 1993) because it was assumed that any and all conversations about STEM-integrated curriculum design would suffice; even if the nature of those conversations resembled discussions typical of the design arena.

The findings from this study therefore indicate the importance of “pushing” active classroom teachers from the design to the mapping arena by instituting curriculum development activities and/or strategies (i.e., processes) that might help a TDT develop a “voice” (Remillard, 2011) or “value” (Dorst, 2011) for the curriculum under development. The curriculum development process will likely be riddled with problems if a TDT solely remains within the design arena; thereby failing to agree on a desired endpoint and value for the curriculum being developed. This will in turn be further confounded when members of the TDT attempt to apply previously successful lesson planning strategies to solve the emergent design problems that surface, which unfortunately require a set of skills (e.g., problem structuring) few teachers possess (Pintó, 2005). Simply put, it would have been beneficial for both TDTs to express their personal beliefs, perceptions, and understandings of the curriculum they were designing (Remillard, 2005) within the mapping arena rather than letting them discuss and dissect content standards and lesson ideas in the design arena.

### Navigating and supporting movement between arenas

In responding to the study’s second research question, the findings presented here highlight the importance of providing *supports* that help teachers navigate from the design arena to the mapping arena (Remillard, 1999).

#### Moving into the mapping arena

Developing curriculum within the mapping arena is not accomplished by finding and modifying possible lesson ideas for upcoming classroom use; a behavior representative of the design arena. Equally involving teachers as partners during the inauguration of curricular change and asking them to work within the mapping arena means teachers need to be prepared to take on an ill-structured design problem with no predetermined pathway to success (Jonassen, 2000). As teachers develop curriculum within the mapping arena, they will begin to form a “voice” other classroom teachers will eventually hear and echo in their own classroom, which brings with it additional pressures (Brown and McIntyre, 1978; Kelly and Staver, 2005; Powell and Anderson, 2002; Shawer, 2010). Ultimately, if TDTs are going to discover a message truly worth delivering, then they should take ownership over the fact they can now actively shape the contents of that message, and this responsibility and challenge is best handled within the mapping arena.

If members of a TDT are willing to reveal their interpretations, perceptions, and beliefs about the conceptual ideas embedded within the curriculum being developed, they will become better teachers and curriculum designers (Voogt et al. 2011). This willingness will also benefit the curriculum being developed (Remillard, 2005). As Remillard (2011) points out, when conversations of this nature take place, the writers of a curriculum begin to develop a “voice that is manifested through the way they communicate” (p. 112) with the teachers that will eventually read, interpret, and use that curriculum. Encouraging TDT members to respond to and participate with the views they are promoting will allow them to “respond directly to the curriculum as a subjective scheme” (Remillard, 2005; p. 237) and to make their “individual interpretations and decisions explicit to themselves and others” (ibid; p. 239). In sum, if teacher intuition alone informs the curriculum development process, it will limit how teachers engage with the teacher-curriculum participatory relationship (Remillard, 2005). Teachers are not typically asked to divulge, discuss, and instill their personal beliefs about the value of integrated learning experiences in the design arena so it is unlikely a worthwhile message will be revealed and incorporated into the curriculum.

### Implications

Involving teachers who do not fully embrace the nature of the design work required for full-scale curriculum development within the mapping arena will likely result in the creation of curricular resources few will want to use because it was developed by individuals who were unaware of the commitment needed to engage in the process. Simply put, some teachers might decline to participate in curriculum development projects once informed the task includes prescribing what happens in someone else’s classroom because the curriculum resources being produced, as cultural artifacts, have the power to afford and constrain human activity in the classroom (Brown, 2009; Remillard, 2011; Wartofsky, 1973); a responsibility not to be taken lightly. Rather than just believing that most teachers will be successful members of a TDT, future research should investigate the characteristics and dispositions (e.g., Goel and Pirolli, 1992) that align well with the design work necessitated via STEM-integrated curriculum design.

Finally, if teachers are going to be successful curriculum designers, it might be necessary for them to learn about alternative skill sets (e.g., Ertmer et al. 2008) or frameworks (Hong and Choi, 2011) that get used and drawn upon by expert designers. Expert designers, for example, as opposed to engaging in “brainstorming” sessions often divert their energy towards identifying *frames* that might link the potential known variables and unknown variables in the generic equation: WHAT + HOW = VALUE. Discovering a new frame is not accomplished directly, but rather tangentially by searching for clues within the “broader problem context” (Dorst, 2011). The many contexts, concepts, and “themes” of STEM-integrated curriculum design could similarly be searched for clues via purposeful discussions (Binkhorst et al. 2015) with the aim being to discover new frames “that allow the central paradox to be approached in a new and interesting way” (Dorst, 2011; p. 528). Ultimately, if innovative learning experiences that make sense pragmatically are going to be included in STEM-integrated curriculum, it might be necessary to *drive* teachers towards moments of “insightful invention, discovery and disclosure” (Dorst, 2011; p. 528) rather than just expecting it will happen.

## Additional file

Additional file 1: Three-way contingency table, selective coding results. (DOCX 15 kb)
