# Supplementary material for: Exploring teacher design team endeavors while creating an elementary-focused STEM-integrated curriculum
Source: Int J STEM Educ. 2017 Oct 6;4(1):21. doi: 10.1186/s40594-017-0084-1 (PMC6310377; doi:10.1186/s40594-017-0084-1)
Supplement: Supplementary file 1 — Three-way contingency table, selective coding results. (DOCX 15 kb) [file 40594_2017_84_MOESM1_ESM.docx]

Additional file 1: Three-way contingency table, selective coding results

|  | 1. Ambiguous Specification of Goals | 2. Need to integrate multiple knowledge domains | 3. No Known Solution Path |
| --- | --- | --- | --- |
| A. design problem structuring | 32 | 9 | 50 |
| B. modularity decomposition | 26 | 7 | 31 |
| C. distinct problem solving phase | 32 | 7 | 42 |
| D. incremental development of artifact | 25 | 12 | 19 |
| E. artificial symbol systems | 22 | 5 | 18 |
| F. reversing direction of transformation function | 18 | 12 | 18 |
| G. abstraction hierarchies | 11 | 6 | 4 |
| H. control structure | 11 | 7 | 13 |
| I. making and propagating commitments | 27 | 9 | 43 |
| J. personalized stopping rules and evaluation functions | 57 | 25 | 63 |
| K. predominance of memory retrieval | 19 | 6 | 23 |
| L. constructing and manipulating models | 4 | 1 | 2 |
